# Examples of Outcome Reporting Bias in Vaccine Studies: Illustrating How Perpetuating Medical Consensus Can Impede Progress in Public Health

**DOI:** 10.7759/cureus.29399

**Published:** 2022-09-21

**Authors:** Gary S Goldman

**Affiliations:** 1 Research, Independent Computer Scientist, Bogue Chitto, USA

**Keywords:** varicella vaccine, vaccines, thimerosal, public health, outcome reporting bias, herpes zoster, mmr vaccine, medical consensus, infant mortality rates, autism spectrum disorder (asd)

## Abstract

Introduction: Outcome reporting bias in vaccine studies is a widespread problem among all researchers who have a tendency to report selective results and conclusions that support their beliefs and values or those of sponsoring agencies. Especially during the COVID-19 pandemic, this bias surfaced through the unprecedented proliferation of conflicting vaccine studies. Many researchers strongly recommend and report on the safety and effectiveness of the COVID-19 vaccine. Those researchers who embrace the COVID-19 vaccine and vaccines, in general, are often dismissive of other researchers who present views that differ from medical orthodoxy and oppose medical consensus.

Methods: The aim of this analysis is to critically evaluate seven vaccine studies using qualitative and/or quantitative approaches to identify outcome reporting bias and assess its potential impact on the stated conclusions that align with medical consensus. Four studies claim to have found no association between autism and (a) blood levels of mercury, (b) measles, mumps, and rubella (MMR) vaccine, and (c) thimerosal-containing vaccines. Three other studies claim no association exists between infant mortality rate and the number of vaccine doses, universal varicella vaccination and herpes zoster, and pandemic influenza vaccines and fetal losses.

Results: The presence of outcome reporting bias and independent reanalysis demonstrated an impact on both the direction and magnitude of the observed effect - raising questions concerning the robustness of the original study design and conclusions and challenging the current medical consensus. Medical consensus has exonerated vaccines as having any causal relationship to autism spectrum disorders (ASDs), yet no other reasonable cause has been proposed. Medical consensus attributes significant ASD increases to better case ascertainment and broadened clinical diagnosis. According to 2018 data, an estimated 1 in 44 eight-year-olds has been identified with ASD. From 1990 to 2019, there have been an estimated two million new cases of ASD in the US, with lifetime social costs exceeding $7 trillion (in 2019 dollars). Can perpetuating medical consensus impede the advancement of public health? Or has it already done so?

Conclusions: Conflicts of interest (e.g., financial) that abound between health regulatory agencies and the pharmaceutical industry impact what is ultimately reckoned as medical consensus. Outcome reporting bias that is inherent to all researchers to some degree, obscures medical and scientific truth. Advancement of public health requires that researchers have integrity and an openness and willingness to collaborate to resolve contradictory findings. In fact, it is usually through meticulous, rigorous, scientific investigation of contradictory findings that medical science has advanced and contributed to improvements in public health - since medical consensus and orthodoxy can be incorrect.

## Introduction

Outcome reporting bias in vaccine studies is a widespread problem among all researchers [1] who have a tendency to report selective results and conclusions that support their beliefs and values or those of sponsoring agencies. Medical journals recognized by the National Library of Medicine (NLM) influence this bias by preferentially publishing studies reporting positive vaccine outcomes. Financial profits accrue to such journals through the publication of articles that promote the paradigm of treatments that support pharmaceutical interventions [2]. This bias has led to selective indexing in the PubMed database/search engine (an NLM resource) and explains why, in part, peer-reviewed journals that focus on nutritional therapeutics, orthomolecular medicine, and research concerning non-pharmaceutical alternatives have failed to garner acceptance by NLM.

Especially during the COVID-19 pandemic has outcome reporting bias surfaced through unprecedented proliferation of conflicting vaccine studies. Many researchers strongly recommend and report on the safety and effectiveness of the COVID-19 vaccine. Those researchers who embrace the COVID-19 vaccine and vaccines in general are often dismissive of other researchers who present views that differ from medical orthodoxy and oppose medical consensus. Those presenting factual data and analyses critical of medical consensus are blamed (censored) for precipitating vaccine hesitancy through "misinformation." 

Marcia Angell (who previously served as an Executive Editor and later as the Editor-in-Chief) of The New England Journal of Medicine and Editor-in-Chief of The Lancet, Richard Horton, both caution that a proliferation of vaccine studies published in prestigious peer-reviewed medical journals possess such an overwhelming degree of outcome reporting bias, that they serve, in large part, as a marketing arm of the pharmaceutical industry [3,4]. Outcome reporting bias inherent to researchers on both sides of the COVID-19 issue is likely responsible for raising notable questions in the public health arena, some of which include the following: Why did Dr. Philip R. Krause, who served for some thirty years at FDA’s Center for Biologics Evaluation and Research (CBER) and in the capacity of Deputy Director of the Office of Vaccines, Research, and Review (OVRR), recently resign prior to the critical period of evaluating COVID booster vaccine efficacy [5]? Why did scientists and medical researchers resort to taking legal action against the FDA to obtain clinical data and documents related to the licencing of the Pfizer-BioNTech Covid-19 vaccine? Can mRNA be reverse transcribed into DNA?

## Materials and methods

Data in the various studies referenced are available from the corresponding study authors or journal editors. The appendices provide referenced email communications obtained through Freedom of Information Act (FOIA) requests, a form letter from a journal editor declining publication, and pertinent extracts from CDC websites.

The methods of analyses vary from study to study, and specific methods are discussed in detail in each referenced study. Where applicable, statistical significance was taken at p < 0.05. Empirical analysis of outcome reporting bias has in some cases involved the methods summarized as follows: Study 1 uses a two-tailed t-statistic to investigate whether there is a statistically significant difference between the blood mercury levels of (a) children with autism spectrum disorder (ASD) and (b) normally developing children. Study 2 computes a rate ratio of children diagnosed with ASD following measles, mumps, and rubella (MMR) vaccination to those children not administered MMR vaccination. A rate ratio greater than 1 indicates an increase in ASD among those vaccinated. Study 3 assesses the discrepancy between statements communicated among authors via email and statements reported concerning study data trends and conclusions. Study 4 notes the impact of a biased dataset and the use of heterogeneous and homogeneous data on linear regression analyses of infant mortality rates (IMRs) of reported nations as a function of the corresponding number of vaccine doses specified in the nation's infant immunization schedule. Study 5 illustrates the importance of analyzing a full dataset instead of a selected sample that is not characteristic of the population. Study 6 qualitatively considers HZ incidence reported over 60 years starting in 1945 and investigates a two-segment, piecewise linear model and the year of its breakpoint to determine if universal varicella vaccination had an impact on increasing HZ incidence rates. Study 7 investigates the number of fetal losses reported annually for each influenza season from 1990/1991 through 2002/2003 and presents unadjusted rates of fetal loss per 1 million pregnant women vaccinated as well as an ascertainment-corrected rate derived through the use of capture-recapture statistics. A comparison was made of fetal-loss reports in influenza seasons both before and after a large spike in fetal loss reports that occurred during the influenza pandemic of 2009/2010.

The aim of this analysis is to critically evaluate seven vaccine studies using qualitative and/or quantitative approaches to identify outcome reporting bias and assess its potential impact on the stated conclusions that align with medical consensus. These studies claim to have found no association between (1) blood levels of mercury and autism, (2) MMR vaccine and ASDs, (3) thimerosal-containing vaccines and autism incidence rates, (4) number of vaccine doses and IMRs, (5) MMR vaccine and pervasive development disorders (PDDs), (6) universal varicella vaccination and herpes zoster (HZ), and (7) pandemic influenza vaccines and fetal losses.

## Results

After each study was critically evaluated by one or more independent researchers, reanalysis yielded results that challenged medical consensus and orthodoxy, thus confirming Nickerson’s maxim, "Many beliefs may be held with a strength or degree of certainty that exceeds what the evidence justifies." [6]

Study 1: Association between blood levels of mercury and autism

In 2004, Ip et al. initially reported no statistically significant (*p* = .15) difference between the blood mercury levels in 82 children with ASD and 55 normally developing children [7]. This result contributed to the conclusion that there was "no causal relationship" between mercury (thimerosal) in vaccines and autism. Upon reanalysis by DeSoto and Hitlan, an error in the original statistical analysis revealed a significant relation (*p* = .024) did exist between blood mercury levels and autism using a two-tailed t-test for a non-directional hypothesis [8]. Ip et al. subsequently conceded the presence of statistical flaws and published an erratum that reported an updated two-tailed statistic of *p* = .056.

Study 2: Association between MMR vaccine and ASD

A 2002 study by Madsen et al. consisting of 537,303 Denmark children, representing 2,129,864 person-years, of which 82% had received the MMR vaccine, reported an 8% lower risk of an ASD in the group of vaccinated as compared to unvaccinated children [9]. Thus, it was concluded, "This study provides strong evidence against the hypothesis that MMR vaccination causes autism." [9]

**Figure 1:**
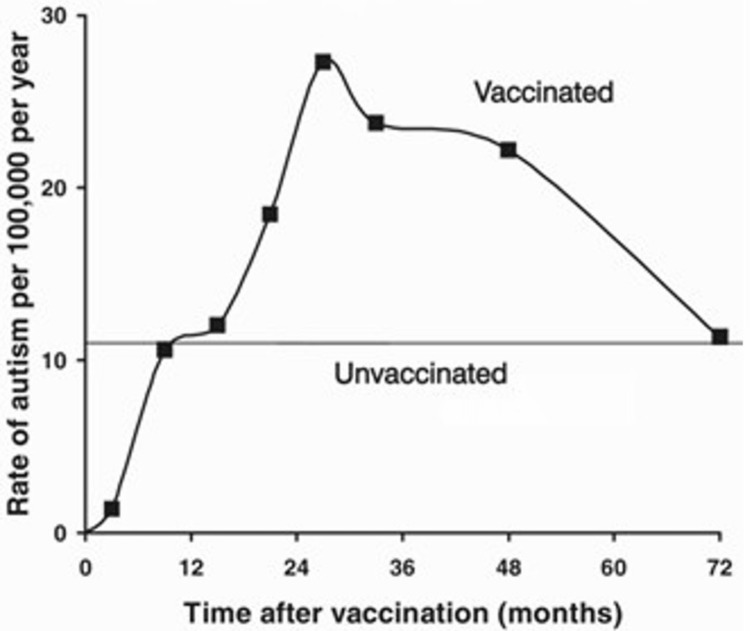
Annual incidence rate of autism in Denmark by time after vaccination, compared to overall annual rate of 11/100,000 for the no-vaccination group. Source. S. Suissa, Dept. of Epidemiology and Statistics, McGill University (Suissa granted permission for reproducing the figure).

However, many Denmark children in the Madsen et al. study from January 1991 through December 1998 were too young to have been diagnosed with autism, such that the number of autistic children were underrepresented. Dr. Samy Suissa, Department of Epidemiology and Statistics at McGill University, reanalyzed the Madsen data, investigating the rate of autism by the time of vaccination, and found a 45% greater risk of ASD in the vaccinated vs. unvaccinated children. The New England Journal of Medicine declined to publish Dr. Suissa’s November 10, 2002, Letter to the Editor, which included the following criticism of the Madsen et al. study:

Madsen et al. observed an adjusted rate ratio of autistic disorder after vaccination of 0.92 relative to no vaccination when the crude rate ratio (my computation) was 1.45 (95% confidence interval 1.08-1.95). Moreover, the rate by time since vaccination increases to a high of 27.3 two years after vaccination (rate ratio 2.5) and decreases thereafter to 11.4 per 100,000 per year (Figure 1). Although it is stated that age adjustment eliminated these rate increases, the corresponding data are unusual. Indeed, the rates of autistic disorder by age at vaccination, although not the age at follow-up, are 18.9, 14.8, 24.6, 26.9, and 12.0 per 100,000 per year, respectively, for the age of 35 months. These rates are all above the overall rate of 11.0 for the reference group of no vaccination, over all ages. It is then somewhat implausible for the adjusted rate ratio to fall below 1 unless the risk profile by age in the unvaccinated is vastly different than in the vaccinated (effect-modification). In this case, the adjustment for age could have been artificial. It would then be useful to present rates on subjects 24-29 months since vaccination and on the unvaccinated (crude rate ratio of 2.5) stratified by age. Otherwise, one could be tempted to conclude that the figure is in fact suggestive of an association between MMR vaccination and the risk of autism.

Publication of Dr. Suissa’s letter to the editor, even if his calculations were erroneous, would have promoted transparency and important dialogue that could have contributed to establishing the integrity of Madsen’s conclusions.

Study 3: Association between thimerosal-containing vaccines and autism

Consider yet another study by lead author Madsen that, this time, investigated a potential correlation between thimerosal and autism. In an email communication dated November 11, 2002, obtained via FOIA request, Marlene Lauritzen (the second listed study author) writes to other co-authors, Poul Thorsen, Kreesten Madsen, and Diana Schendal, regarding their study [10], "I need to tell you that the figures in the manuscripts *do not include the latest data from 2001* [emphasis added]. I only have these figures as a paper version and they are at work…. But the incidence and prevalence are *still decreasing* [emphasis added] in 2001" (Appendix I). Given this internal email shared between study authors, known data were omitted, leading to a false conclusion that the discontinuation of thimerosal-containing vaccines in Denmark in 1992 resulted in an increase in autism incidence. The authors deceptively concluded, "Our ecological data do not support a correlation between thimerosal-containing vaccines and the incidence of autism" [10]. Gina Steiner of the American Academy of Pediatrics (APP) promoted this study's fabricated conclusion in a news brief (Appendix II).

Study 4: Association between IMR and number of vaccine doses

Nysetvold et al. (hereinafter referred to as Bailey’s rebuttal) [11] target a 2011 study by Miller and Goldman for reanalysis because it reports a finding contrary to medical consensus - a statistically significant, positive correlation (*r* = 0.70, *p* < .0001) demonstrating that as nations require more vaccine doses for their infants, IMR increases (worsens) [12].

Since medRxiv does not accept rebuttals as a general policy, it is an apparent double standard on the part of medRxiv staff to accept the Bailey rebuttal (initially including libelous/defamatory language and numerous statements demonstrating outcome reporting bias) but reject the preprint of Goldman and Miller [13], addressing the violations of Nysetvold et al. of the scientific method that included confounding analyses arising from heterogeneous data and IMR instability.

The Bailey rebuttal, in reality, is a new analysis using selective data to seemingly prove a preconceived conclusion - that all vaccines are "safe and effective," and more specifically, to debunk the counterintuitive positive correlation reported by Miller-Goldman.

Bailey and coauthors baselessly claim that the findings of Miller and Goldman are due to "inappropriate data exclusion" by failing to analyze the "full dataset" of all 185 nations. This statement is demonstrably false. Miller and Goldman sought to investigate why the US (which specifies the most vaccine doses of any nation) ranks 34th among nations ranked in ascending IMR order. Four of these 34 nations were excluded on the basis of IMR instability (i.e., nations reporting five or fewer infant deaths), leaving 30 nations for analysis that all had vaccination rates >90% and possessed homogeneity of socioeconomic factors.

**Figure 2:**
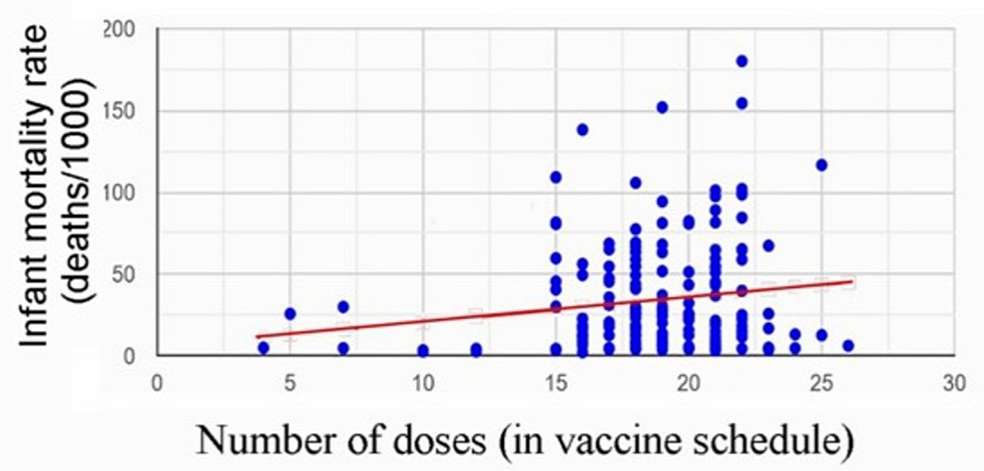
Best-fit line of IMR vs number of vaccine doses from the linear regression analysis performed in the Bailey rebuttal using 185 nations. IMR: infant mortality rate [11].

The Bailey rebuttal analyzes the "full dataset" of 185 nations (Figure 2) and reports a small, statistically significant positive correlation, *r* = 0.16 (*p* < .026) and concludes, "the positive correlation between IMR and immunization schedules disappeared…" and "this indicates that there is no relationship…" [11]. These statements demonstrate the outcome reporting bias that arises from the flawed methodology of analyzing nations with varying vaccination rates and heterogeneity of socioeconomic factors. The Bailey rebuttal indiscriminately aggregates highly developed nations with Third World nations experiencing numerous substandard socioeconomic and environmental conditions so as to obscure any impact of the number of vaccine doses on IMR.

Publication bias explains why both peer-reviewers and medical journal editors may ignore selective and misleading data analyses of Nysetvold et al. and accept Bailey’s rebuttal on the basis that (1) its overall theme aligns with medical consensus by promoting that vaccines are "safe and effective," (2) it discusses the role that misinformation plays in vaccine hesitancy (i.e., a current topic of interest to readers), and (3) its acceptance generates journal revenue of $1,800 or more.

Study 5: Association between MMR vaccine and PDD

Fombonne et al. investigated PDD in Montreal, Quebec, Canada among 27,749 English-speaking students born from 1987 to 1998, attending 55 schools [14]. In reality, selection bias was present in this study, which included 14% of students attending schools in the Lester B. Pearson School Board. The study reported PDD rates significantly increased when MMR vaccination rates decreased. This selective study cohort represented the City of Quebec, and the reported statistics were not applicable to the City of Montreal or the Province (located 157 miles, or 265 km, away), where PDD rates significantly increased in five school boards when MMR vaccination uptake rates also significantly increased. Dr. David Ayoub obtained a more complete dataset (i.e., the remaining 86% of the available data) that implicated vaccines, and not better diagnosis or broadened diagnostic criteria, in the significant increase in childhood autism. His Letter to the Editor of Pediatrics was declined.

Study 6: Association between universal varicella vaccination and HZ

**Figure 3:**
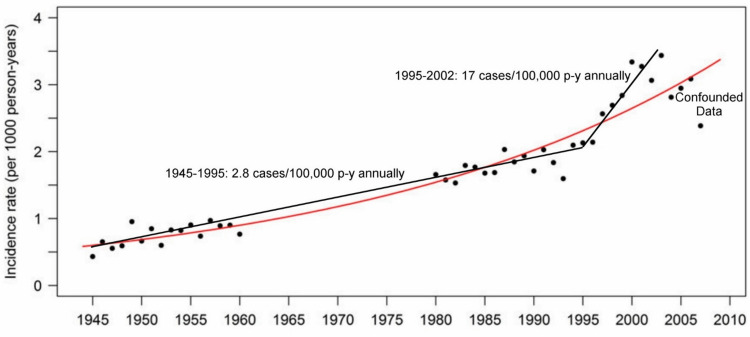
Herpes zoster incidence rate by year, 1945-2007, comparison of trend lines. Red curve: trend reported by Kawaii et al. [15]. Black lines: trend reported by Goldman [17] (excluding HZ incidence rate among varicella-vaccinated children).

An "Editor’s Choice" article published in Clinical Infectious Disease (CID) by Kawai et al. reported, "no change in the rate of [herpes zoster] increase before vs. after the introduction of the varicella vaccination program" [15]. However, Figure 3 presents an alternative analysis (of Kawai’s Figure 1) that supports a different, plausible conclusion that corroborates Dr. Hope-Simpson’s 1965 hypothesis: "The peculiar age distribution of zoster may in part reflect the frequency with which the different age groups encounter cases of varicella and, because of the ensuing boost to their antibody production, have their attacks of zoster postponed." [16] Multiple studies support that exposure to children shedding wild-type varicella-zoster virus (VZV) boosts immunity in adults to suppress the reactivation of VZV as HZ. Thus, a decline in exogenous exposures through universal varicella vaccination might have an effect on increasing HZ incidence among adults [17].

Figure 3 shows an unusual downward trend when we confine our inspection to the period of 1999-2007. There is an anomaly (not addressed by the authors) in the dataset whereby the last four data points corresponding to 2004-2007 (approximately 9 to 12 years post-varicella vaccine licensure) demonstrate a lower HZ incidence rate relative to 2001. Also, it is unusual that the HZ incidence rate corresponding to the last 2007 data point is lower than the 1997 HZ incidence rate ten years earlier. These last four data points are confounded as the result of the influence of a relatively low HZ incidence rate among the increasing numbers of varicella-vaccinated children that have been averaged with the increasing HZ incidence rate among adults [17]. Thus, Figure 3 demonstrates two distinct piecewise linear trends with a breakpoint in 1995 (the year of varicella vaccine licensure): an HZ incidence rate that changes from 2.8 cases/100,000 person-years (p-y) annually for the period of 1945 to 1995, to 17 cases/100,000 p-y annually for the period of 1995-2002 (and thereafter, when the lower HZ incidence rate among varicella-vaccinated children is excluded). While there was no apparent change in the rate of increase for the population as a whole before versus after the introduction of the varicella vaccination program, the significant increase in HZ incidence among adults post-licensure was masked by a concomitant decrease in HZ incidence among children. A Letter to the Editor of CID sharing this reanalysis was promptly declined (Appendix III).

Study 7: Unusual pattern in fetal losses following pandemic influenza vaccines

Utilizing data from the Vaccine Adverse Event Reporting System (VAERS), Moro et al. [18] established that during 19 influenza seasons (1990/1991 through 2008/2009), there were a total of 17 spontaneous abortions (SABs) and 6 stillbirth reports following trivalent inactivated influenza vaccine, yielding a rate of "1.9 [fetal losses] per 1 million (or 23/11,800,000) pregnant women vaccinated." It is noteworthy that because VAERS is a passive reporting system, fetal-loss reports are grossly underreported; thus, this raw, unadjusted rate was underestimated by one or more orders of magnitude. This VAERS-derived rate was an outcome reporting bias designed to mislead both physicians and patients to believe fetal loss due to influenza vaccine was negligible and to quell concerns leading to vaccine hesitancy - even though the pandemic season was unique and for the first time required the administration of not one, but two (pandemic and seasonal) influenza vaccines.

**Figure 4:**
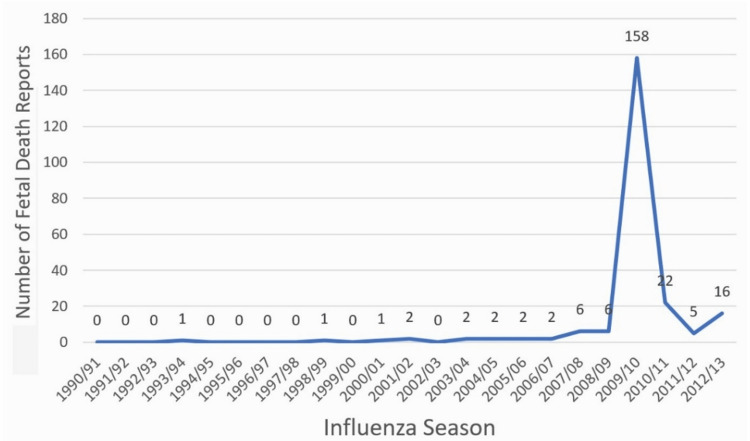
Updated number of fetal death reports (from VAERS) during each influenza season. VAERS: Vaccine Adverse Event Reporting System.

Outcome reporting bias might explain why the CDC-sponsored study by Moro et al. chose to consider data through 2008/2009 [18] and stopped short of the pandemic influenza season of 2009/2010 when both the pandemic A-H1N1 and seasonal influenza vaccines were administered to pregnant women during any stage of pregnancy and an unusually large number of fetal losses had been reported to VAERS (Figure 4). On October 28, 2010, Dr. Tom Shimabukuro, Deputy Director of the H1N1 Vaccine Task Force at the CDC, shared an extra "slide #20" in a public presentation at an Advisory Committee on Immunization Practices (ACIP) meeting in Atlanta, Georgia. This slide detailed a total of 170 VAERS fetal-loss reports, i.e., 149 SAB and 21 stillbirths reported between October 1, 2009, and June 30, 2010. During the month prior to Dr. Shimabukuro’s presentation, a seemingly contradictory report was given by Dr. Marie McCormick, Chairperson of CDC’s H1N1 Vaccine Safety Risk and Assessment Working Group, to the Department of Health and Human Services (HHS) at meetings of the Advisory Commission on Childhood Vaccines (ACCV) held September 3, 2010, in Rockville, Maryland, and the National Vaccine Advisory Committee (NVAC) held September 10, 2010. She testified that neither any unusual signals nor adverse outcomes had occurred among pregnant women vaccinated during the 2009/10 pandemic season based on a review of several databases, including VAMPSS (Vaccine and Medications in Pregnancy Surveillance System) and VSD (Vaccine Safety Data Network).

In 2009, the CDC’s ACIP recommended pregnant women "get the seasonal flu shot and the 2009 H1N1 flu shot" on the basis that (1) the seasonal influenza vaccine was associated with low fetal-loss reports to VAERS occurring during the prior 19 single-dose seasons and (2) the pandemic A-H1N1 vaccine "is made in the same way and in the same places as the seasonal flu shot" (Appendix IV). This qualitative assessment of safety was subject to outcome reporting bias since it overlooked the fact that during the first 14 of the prior 19 seasons, a more precautionary vaccination approach prevailed during which only those pregnant with special circumstances or those beyond their first trimester were vaccinated. Moreover, despite the majority of seasonal and pandemic influenza vaccines each containing 25 mcg of mercury, no warning or concern was issued by the CDC regarding pregnant women receiving up to 50 mcg of mercury - resulting in an *in utero* mercury exposure to the developing fetus that could potentially exceed the FDA safety level (i.e., reference dose of 0.0001 mg/kg/day or 0.1 mcg/kg/day for ingested mercury) [19].

Later, in another study, Moro et al. concluded [20], "the reporting rates to VAERS of SABs and stillbirths were several orders of magnitude lower than the expected rates of fetal loss in the general population of pregnant women…" This misleading statement on vaccine safety again derives from outcome reporting bias as a result of the authors' invalidly (improperly) comparing severely underreported fetal-loss reports to VAERS with studies having more complete case ascertainment. VAERS trends over the 19 seasons prior to the pandemic establish a background rate that averages 1.21 fetal-loss reports per season. The pandemic season’s 170 VAERS fetal-losses represent a 140-fold (170/1.21) increase that is two orders of magnitude above the VAERS fetal loss background rate. Using an updated number of reports given in Figure 4, a 26-fold increase occurred from six fetal-loss reports in 2008/09 to 158 reports in the 2009/10 pandemic season. Following the observed spike in fetal-loss cases during the two-dose pandemic season, the CDC recommended a single quadrivalent influenza vaccine that included the A-H1N1 strain (reducing the mercury load by half relative to the pandemic season). There was only a 3.7-fold (22/6) increase in fetal-loss reports from the season immediately before (2008/09) versus after (2010/11) the pandemic (Figure 4).

Two different CDC-sponsored studies by Moro et al. investigating VAERS reports concluded there were "no unusual pattern" [18] and no "concerning patterns of maternal or fetal outcomes" (Figure 4) [20]. Moro et al. postulated that a "Weber-like effect" (i.e., a temporal reporting pattern whereby the number of reported adverse events for a new drug temporarily increases) may be responsible for the spike in fetal-loss reports [20]. Goldman [19] demonstrated that a Weber effect was an unlikely explanation and hypothesized that the combined doses of mercury from two influenza vaccines may be responsible for the reported order of magnitude increase in fetal-loss report rates, from 6.8 fetal-loss reports per million pregnant women vaccinated in the single-dose 2008/2009 season to 77.8 (95% C.I.: 66.3-89.4) fetal-loss reports per million in the two-dose 2009/2010 season [20]. Capture-recapture statistical analysis found that only 13.2% of actual fetal-loss cases were reported to VAERS, for an ascertainment-corrected rate of 590 fetal-loss reports per million (or 1 fetal loss per 1,695 pregnant women vaccinated) during the pandemic season [19]. Another study by Brown and Austin [21], which used different methods, corroborated fetal concerns (including death) implicating mercury exposure.

## Discussion

The presence of outcome reporting bias and independent reanalysis of each study demonstrated an impact on both the direction and magnitude of the observed effect - raising questions concerning the robustness of the original study design and conclusions and challenging the current medical consensus. Each of the seven study outcomes experienced a reversal, yielding statistically significant positive correlations between (a) MMR vaccine and ASD (and PDD), (b) thimerosal-containing vaccines and autism, (c) the number of vaccine doses and IMRs, (d) universal varicella vaccination and HZ incidence, and (e) pandemic influenza vaccines and VAERS fetal-loss reports. Once "overwhelming" evidence has accumulated in a given area of research (reinforced by publication bias that serves to suppress studies with undesirable results), the medical consensus is further sustained by the publication of a synthesis of the results of all conducted studies via meta-analyses, such as that exemplified by Taylor et al., titled *Vaccines are not associated with autism: an evidence-based meta-analysis of case-control and cohort studies* [22].

Complicating factors underlying outcome reporting bias

In the US, the American Academy of Pediatrics recommended that thimerosal be removed from routine childhood vaccines in 1999; however, the existing, unexpired doses of thimerosal-containing vaccines continued in use throughout 2002. In 2002, the CDC recommended two new thimerosal-containing influenza vaccines for children aged 6-23 months. Moreover, in 2004, CDC recommended that women, in any trimester of pregnancy, be administered a seasonal influenza vaccine (resulting in an *in utero* mercury exposure to the fetus) [23] since most influenza vaccines contained thimerosal. These new directives, including the adoption of aluminum-containing vaccines [24], served to confound studies that expected ASD and other adverse reactions (including autoimmunity) to subsequently decline following the CDC’s premature declaration in 2001 that thimerosal had been removed from routinely administered childhood vaccines in the US.

Especially during the COVID-19 pandemic, the media has played a key role in stimulating outcome reporting bias concerning vaccine efficacy. This was alluded to by Peter Daszak, President of EcoHealth Alliance, three years prior to the start of the COVID-19 pandemic in February 2016:

Daszak reiterated that until an infectious disease crisis is very real, present, and at an emergency threshold, it is often largely ignored. To sustain the funding base beyond the crisis, he said, we need to increase public understanding of the need for MCMs (medical countermeasures) such as a pan-influenza or pan-coronavirus vaccine. The media is a key driver, and the economy follows the hype. We need to use that hype to our advantage to get to the real issues. Investors will respond if they see a profit at the end of the process, Daszak stated [25].

A more elaborate form of outcome reporting bias in vaccine studies (often sponsored by the pharmaceutical industry) occurs when input data are manipulated until various statistical computer programs output selective results that appear correct, demonstrate statistical significance, and support a predetermined conclusion. Under such a scenario, the COVID-19 vaccine may be purported to be "safe and effective" based on selective data showing a lower incidence of hospitalization and death due to COVID disease among COVID-vaccinated vs. unvaccinated patients. However, a comprehensive risk-benefit assessment may yield public health concerns when data with wide disparity have been properly stratified, or additional data are available for all-cause morbidity and mortality - especially if ascertainment-corrected vaccine-induced events (e.g., reproductive toxicity, miscarriages, micro blood clots or vaccine-induced immune thrombotic thrombocytopenia, cancer, sudden adult death, inflammation of heart, liver, and kidneys, etc.) exceed population background rates for those same events. Consider the nonsteroidal anti-inflammatory drug (NSAID) Vioxx, licensed by the FDA in 1999 for patients experiencing arthritic pain. While Vioxx was initially believed to reduce the risk of serious gastrointestinal complications by 50% relative to the NSAID pain-reliever naproxen, by 2004, Vioxx was withdrawn from the market after studies reported a four-to five-fold increased risk of cardiovascular events (i.e., heart attack and stroke).

Outcome reporting bias prolongs the vaccine/autism debate

Between 2001 and 2007, more than 5,000 families filed autism claims with the federal Vaccine Injury Compensation Program (VICP). In these cases, many parents describe their children as exhibiting late-onset autism-like features shortly following vaccine administration, i.e., children who could perform tasks and follow directions, as well as speak and maintain eye contact, suddenly regress, losing these acquired skills and abilities. Poling et al. document such a case in a 19-month-old female with a pre-existing, previously undiagnosed, mitochondrial disorder that predisposed her vulnerability to the receipt of five vaccines administered in a single physician’s visit against nine vaccine-preventable diseases (i.e., diphtheria, tetanus, and pertussis; Haemophilus influenzae B, measles, mumps; and rubella, polio, and varicella) [26]. Years later, this child’s family received compensation of $1.5 million from VICP and $500,000 per year for care by showing a plausible biological mechanism.

Medical consensus has exonerated vaccines from having any causal relationship to ASD. Gerber and Offit in *Vaccine and Autism: A Tale of Shifting Hypotheses* conclude:

Twenty epidemiologic studies have shown that neither thimerosal nor MMR vaccine causes autism. These studies have been performed in several countries by many different investigators who have employed a multitude of epidemiologic and statistical methods. The large size of the studied populations has afforded a level of statistical power sufficient to detect even rare associations. These studies, in concert with the biological implausibility that vaccines overwhelm a child’s immune system, have effectively dismissed the notion that vaccines cause autism. Further studies on the cause or causes of autism should focus on more promising leads [27].

All epidemiologic studies have methodological limitations. Any study (despite publication in a prestigious journal) whose data are unavailable for independent reanalysis (due to authors classifying the data as confidential or journal editors unwilling to enforce data availability) should be retracted since reported results and conclusions are unverifiable. Despite accolades ascribed to epidemiologic studies (including those "unverified") that endorse medical consensus, we must ask: Do they present the truth so convincingly that researchers should "focus on more promising leads" given the thousands of parents who have reported their children regressing following vaccine administration? Do health regulatory authorities believe that every single one of these late-onset autism cases is merely coincidental and that no evidence of a dose-response effect or plausible biological mechanism exists? Can medical consensus be trusted when it attributes significant ASD increases to better case ascertainment and changes in clinical diagnosis? What are the chances that at least some children have undiagnosed mitochondrial disorders similar to the Poling child discussed earlier and experienced similar vaccine injury?

Studies that rely on epidemiologic methods may not be able to successfully control for confounding and bias due to their inability to distinguish congenital cases of autism (i.e., a genetic etiology) from cases of late-onset autism (i.e., a potential vaccine etiology). Without access to specific childhood immunization records, epidemiologic studies have limited ability to ascertain the potential synergistic effects of various combinations of vaccines. Additional fields of neurology, toxicology, virology, pharmacology, and pharmacokinetics may help medical professionals to better elucidate the pathogenesis of adverse vaccine effects due to exposures to aluminum, thimerosal (ethyl-mercury), and other vaccine components.

Of the many experts who are certain that the MMR vaccine and thimerosal-containing vaccines have no causal relationship to ASD, not one has yet to propose another reasonable cause. Instead, on February 8, 2007, CDC director Dr. Julie Gerberding admitted, "… we can't yet tell if there is a true increase in ASDs or if the dangers are the result of our better studies" [28]. Dr. Gerberding, prior to her leaving the CDC to serve as President of Merck’s vaccine division, has also been widely quoted as admitting: "We don’t know what causes autism; that’s a fact."

Segal and Shoenfeld have proposed a plausible mechanism that is suspected in adverse vaccine reactions [29]: "Molecular mimicry refers to a significant similarity between certain pathogenic elements contained in the vaccine and specific human proteins. This similarity may lead to immune crossreactivity, wherein the reaction of the immune system towards the pathogenic antigens may harm the similar human proteins, essentially causing autoimmune disease."

According to 2018 data, an estimated 1 in 44 eight-year-olds has been identified with ASD, according to the Autism and Developmental Disabilities Monitoring (ADDM) Network. In other terms, Cakir et al. report that from 1990 to 2019, there have been an estimated two million new cases of ASD in the U.S. with lifetime social costs exceeding $7 trillion (in 2019 dollars) [30]. Can perpetuating medical consensus impede the advancement of public health? Or has it already done so?

Limitations

The main limitation of this analysis was that outcome reporting bias was investigated in seven vaccine studies, yet it impacts thousands of vaccine studies and is present to some degree in all research.

## Conclusions

This study examined several examples of outcome-reporting biases that are found in many vaccine studies. Conflicts of interest (e.g., financial) that abound between the FDA, CDC (or foreign entities such as the Danish Staten Serum Institut), and the pharmaceutical industry impact what is ultimately reckoned as medical orthodoxy or scientific consensus. Moreover, regulatory agencies seemingly attempt to control the narrative that vaccines are "safe and effective" through their funding or sponsorship of selective studies that often lack data transparency and, in some cases, cross bioethical boundaries. Where vaccine study data are available, independent researchers experience an astonishing level of censorship by medical journal editors who deny publication when outcome reporting bias in the original published studies is exposed and reanalysis leads to conclusions contrary to the medical consensus. This conduct, as well as the outcome reporting bias that is inherent to all researchers to some degree, obscures medical and scientific truth.

Ideally, if conflicts of interest could be eliminated among vaccine study sponsors, regulatory agencies, and the pharmaceutical industry, the fact that various researchers might obtain contradictory results and conclusions would not be worrisome. It may be the case that two contradictory presentations - the one supporting the medical consensus and the one that is antagonistic - may both be flawed! Advancement of public health requires that researchers have integrity and an openness and willingness to collaborate to resolve contradictory or mixed results. In fact, it is usually through meticulous, rigorous, scientific investigation of contradictory findings that medical science has advanced and contributed to improvements in public health - since medical consensus and orthodoxy can be incorrect. The narrative that COVID-19 vaccines are "safe and effective" is not immune to outcome reporting bias. Those in support of medical consensus lose credibility and ultimately undermine public confidence in vaccine programs by censoring researchers reporting deleterious effects, especially when the numbers of adverse reactions become so prevalent that the medical community can no longer claim they are coincidental or when a given vaccine's risk-benefit ratio is shown to be no longer favorable.

## Appendices

Appendix I. Reproduction of email regarding manuscript about Thimerosal and autism (obtained via Freedom of Information Act request)

From:  Kreesten Meldgaard Madsen [KMM@SOCIAU.DK]

Sent:  Wednesday, November 13, 2002 5:33 AM

To:    Marlene Briciet Lauritsen; Poul Thorsen, Schendel, Diana

Subject:  Re: Manuscript about Thimerosal and autism

Hi Marlene,

[Redacted text…] I am not currently at the university but I will contact you and Poul tomorrow to make up our minds.

Best regards.

Kreesten

-----Original Message-----

From: Marlene Briciet Lauritsen [mailto:mbl@dadlnet.dk]

Sent: Wed 13-11-2002 09:24

To: Poul Thorsen, Kreesten Meldgaard Madsen;

Cc:

Subject: Manuscript about Thimerosal and autism

Dear Poul, Kreesten and Diana Schendel

     Attached I send you the short and long manuscript about Thimerosal and autism in Denmark. [Redacted text…]

     I need to tell you that the figures in the manuscripts do not include the latest data from 2001. I only have these figures as a paper version and they are at work [redacted text]. But the incidence and prevalence are still decreasing in 2001. [Redacted text…]

     I look forward to hear from you again.

Best regards

Marlene

Appendix II. Email content of news brief from Gina Steiner to Kreesten Meldgaard Madsen

-----Oprindelig meddelelse-----

Fra: Gina Steiner [mailto:]

Sendr: 15. August 2003 11:53

Til: Kreesten Meldgaard Madsen

Emne: Re: SV: a “news brief” on your study in Pediatrics

Dear Dr. Madsen,

Below is the “news brief” we are planning to send to reporters in about 10 days. I need your approval or comments by this Monday. Because of the time difference, could you look at it by Sunday?

Thank you so much.

Sincerely,

Gina Steiner

American Academy of Pediatrics

846-434-7872

------------

A study of nearly 1,000 Danish children diagnosed with autism over three decades showed no correlation between thimerosal-containing vaccines and the incidence of autism.  According to “Thimerosal and the Occurrence of Autism? Negative Ecological Evidence From Danish Population-Based Data,” autism rates continued to increase after the removal of thimerosal from vaccines in 1992. These increases occurred among children born both before and after the discontinuation of thimerosal use. The study looked at all children between 2 and 10 years old who were diagnosed with autism from 1971-2000, according to the Danish Psychiatric Central Research Register.

[For an interview on this study, please contact Kreesten M. Madsen, MD, at kmm@dadlnet.dk or Marlene B Lauritsen at mbl@psykiatri.aaa.dk (note: the authors are located in Denmark).]

Appendix III. Form letter response declining to publish letter to the Editor

Re:  CID CID-115011, "Letter to Editor regarding Kawai et al. by Gary S. Goldman, PhD"

Dear Dr. Gary Goldman:

Thank you for submitting your Correspondence to Clinical Infectious Diseases. Unfortunately the Editor did not assess your manuscript to have sufficient priority to warrant in-depth review by CID.

We receive a large number of excellent manuscripts. Due to increasing numbers of submissions and the limited space in our journal, we are forced to reject many papers of high quality. We use our best judgment to assess such issues as the quality of the manuscript, its appropriateness for the journal and its level of interest to our general readership. 

We appreciate your interest in CID.

Sincerely,

[***Redacted***], Deputy Editor

Clinical Infectious Diseases

email: 

Appendix IV. Extracts from Centers for Disease Control and Prevention Websites

H1N1 Flu

Available online at: https://www.cdc.gov/h1n1flu/guidance/pregnant.htm

What can I do to protect myself, my baby, and my family?

Getting a flu shot is the single best way to protect against the flu. Talk with your doctor about getting a seasonal flu shot and the 2009 H1N1 flu shot. You will need both flu shots this year to be fully protected against the flu. You should get both shots as soon as they are available to protect you and your baby. The seasonal flu shot has been shown to protect both the mother and her baby (up to 6 months old) from flu-like illness.

Is it safe for pregnant women to get a flu shot?

The seasonal flu shot has been given to millions of pregnant women over many years. Flu shots have not been shown to cause harm to pregnant women or their babies. The 2009 H1N1 flu shot is made in the same way and in the same places as the seasonal flu shot. It is very important for pregnant women to get both the seasonal flu shot and the 2009 H1N1 flu shot.

2009 H1N1 Influenza Vaccine and Pregnant Women:  Information for Healthcare Providers

Available online at https://cdc.gov/h1n1flu/vaccination/providers_qa.htm

Is the 2009 H1N1 monovalent flu vaccine safe for pregnant women?

Flu vaccines have not been shown to cause harm to a pregnant woman or her baby. The seasonal flu shot has been recommended for pregnant women for many years. The 2009 H1N1 monovalent flu vaccine will be made using the same processes as the seasonal flu vaccine, and clinical trials of H1N1 monovalent vaccine safety in non-pregnant children and adults found results similar to those seen in studies of the seasonal flu vaccine. Studies that test the 2009 H1N1 monovalent flu vaccine in pregnant women began in September.

Does the 2009 H1N1 monovalent flu vaccine have preservative in it?

Multi-dose vials of flu vaccine contain the preservative thimerosal to prevent bacterial growth. There is no evidence that thimerosal is harmful to a pregnant woman or a fetus. However, because some women are concerned about exposure to preservatives during pregnancy, manufacturers are producing preservative-free seasonal flu vaccines and 2009 H1N1 monovalent flu vaccines in single-dose syringes. The CDC recommends that pregnant women receive the flu vaccine with or without thimerosal.

Can the 2009 H1N1 monovalent flu vaccine be given at any time during pregnancy?

The seasonal flu vaccine is recommended for all pregnant women at any time during pregnancy and has not been shown to cause harm to a pregnant woman or her baby. The Advisory Committee on Immunization Practices also recommends that the 2009 H1N1 monovalent flu vaccine be given to all pregnant women at any time during pregnancy.
